# Development of a core outcome set for traditional Chinese medicine for atrial fibrillation 

**DOI:** 10.3389/fphar.2026.1731405

**Published:** 2026-05-07

**Authors:** Chenyao Zhang, Zhongping Hao, Danni Li, Jie Wan, Hui Wang, Chunxiang Liu, Lujia Cao, Menglong Shi, Wentai Pang, Xinyao Jin, Bairu Cheng, Jia Qi, Junhua Zhang, Bo Pang

**Affiliations:** 1 Tianjin University of Traditional Chinese Medicine, Tianjin, China; 2 Chinese Core Outcome Sets Research Center, Tianjin, China; 3 Department of Cardiology, Dongfang Hospital Beijing University of Chinese Medicine, Beijing, China; 4 Dongzhimen Hospital, Beijing University of Chinese Medicine, Beijing, China

**Keywords:** atrial fibrillation, core outcome set, delphi survey, methodology, traditional Chinese medicine

## Abstract

**Objectives:**

The inconsistent reporting of clinical trial results in patients with atrial fibrillation (AF) hinders the comparison of findings. Developing a core outcome set (COS) in studies evaluating traditional Chinese medicine (TCM) for AF and recommending measurement instruments and time points may help standardize the selection, reporting, and measurement of outcomes in clinical trials.

**Methods:**

Literature and registered trials were retrieved to systematically collect outcomes. Physician questionnaire surveys and semi-structured patient interviews were conducted to collect outcomes of clinical interest. These outcomes were standardized and compiled into a preliminary outcome pool. Consensus criteria were established in advance. Through two rounds of Delphi surveys and consensus meetings, perspectives from multiple stakeholder groups were gathered to establish a COS for TCM for AF (COS-TCM-AF).

**Results:**

A total of 87 individuals participated in the Delphi survey, with 70 completing both rounds. During the consensus meeting, 19 stakeholders determined that seven core outcomes should be involved in the COS-TCM-AF, including AF episode frequency, AF episode duration, AF burden, TCM symptom-palpitation, thromboembolic (TE) event rate, AF recurrence rate, and incidence of acute heart failure (HF)/acute exacerbation of chronic HF. Among these, AF episode frequency and AF episode duration applied only to patients with paroxysmal AF.

**Conclusion:**

This COS comprehensively addresses multiple aspects including AF episodes, patient symptoms, complication risks and long-term prognosis. It also recommends measurement instruments and time points. Its implementation will contribute to facilitating the comparison of similar studies and provide a reference for selecting and measuring outcomes.

## 1 Introduction

Atrial fibrillation (AF), the most common clinical arrhythmia, exhibits an age-related increasing trend in incidence and has become a key disease affecting cardiovascular health in the elderly population (Wang et al., 2024; Li et al., 2022; Zimetbaum, 2017). Epidemiological surveys indicate that approximately 59 million people worldwide currently suffer from AF, with an incidence rate as high as 12% among individuals aged 80 and above (Linz et al., 2024; Roth et al., 2020). Characterized by chaotic electrical activity in the atria, AF is closely associated with increased all-cause mortality, sudden cardiac death, heart failure (HF), and ischemic stroke (Odutayo et al., 2016; Ruddox et al., 2017; Waldmann et al., 2020). As population ages, AF is projected to impose an increasingly heavy healthcare burden in the future(Guo et al., 2015).

According to its manifestations, AF can belong to the category of “palpitation” in traditional Chinese medicine (TCM). TCM has accumulated extensive clinical experience in treating AF, and an increasing number of studies have shown its notable efficacy in alleviating symptoms such as palpitations and chest tightness, reducing recurrence rates, and improving patients' quality of life (Wang et al., 2022; Lu et al., 2024; Wang et al., 2021). However, current clinical research on TCM for AF faces notable limitations: The lack of standardized selection criteria for outcomes leads to fragmented evidence, while research design flaws and inconsistent reporting of outcomes result in uneven evidence quality. These issues severely impede the effective translation of research evidence into clinical practice, causing inefficient utilization of substantial research resources and constraining the evidence-based development of TCM in treating AF (ZHANG, 2022; Nan Li, 2021; Xinyue Dai, 2022).

Core outcome sets (COS) serve as the recognized minimum set of outcomes to be reported in clinical trials, enabling the comparison and integration of similar research findings to reduce the waste of research resource (Clarke and Williamson, 2015; Chan et al., 2014). Given the current research landscape, this study aims to address these limitations by establishing a COS for TCM treatment of AF (COS-TCM-AF) and by recommending measurement instruments and time points. This may enhance the practical applicability and clinical representativeness of outcomes in TCM for AF research, and build evidence-based support for TCM in the management of AF.

## 2 Methods

This COS was developed following the standardized methodological framework and procedural recommendations provided in the COMET Handbook (Version 1.0) (Williamson et al., 2017). This study adopted a stepwise core outcome set development design. First, outcomes were identified from published studies and registered trials to capture outcomes already reported in the literature. Second, clinician surveys and semi-structured patient interviews were conducted to identify outcomes considered important by stakeholders and to supplement outcomes that may not have been adequately represented in published studies. Third, all identified outcomes were standardized and merged into a preliminary outcome pool, which was then prioritized through Delphi surveys and finalized at a consensus meeting.

### 2.1 Research registration

The working group registered the study on the ChiCOS platform in 2024 (Registration No.: CHICOS2024000033) and obtained approval from the Ethics Review Committee of Tianjin University of Traditional Chinese Medicine (Approval No.: TJUTCM-EC20240010).

### 2.2 Participants

#### 2.2.1 Steering committee and working group

The steering committee comprised 10 members, including clinical physicians, methodological experts, journal editors, and clinical researchers. The steering committee offered professional guidance and consultation, monitored and evaluated the research process, and proposed measures for improvement and recommendations. A working group consisting of 13 members was established to carry out specific tasks, including literature screening, outcome extraction, and clinical research.

#### 2.2.2 Stakeholders

To ensure regional representativeness among physicians participating in the clinical research, we invited clinicians from tertiary hospitals across various regions nationwide to complete the online questionnaire survey. Outpatients and inpatients were invited to participate in semi-structured interviews. As recommended by COS-STAD, COS users, healthcare professionals, and patients are indispensable key participant groups in the Delphi survey process, playing a central role in advancing research progress and ensuring the scientific rigor and practical applicability of outcomes (Kirkham et al., 2017). Participants in the Delphi questionnaire survey for this study included: (1) Cardiologists, encompassing TCM physicians, western physicians and integrative medicine physicians; (2) Patients with non-valvular AF and their caregivers; (3) Pharmaceutical representatives; (4) Clinical research methodologists; (5) Journal editors.

### 2.3 Information sources

#### 2.3.1 Information retrieval

Computerized searches were conducted in both Chinese and English databases. Chinese databases included CNKI, Wanfang, VIP, and SinoMed, while English databases comprised PubMed, Embase, Cochrane Library, and Web of Science. We also searched the Chinese Clinical Trial Registry (ChiCTR) and ClinicalTrials.gov databases for all studies involving AF. The search period spanned from inception to 30 June 2024 (Supplementary Table S1). No language limits were applied.

#### 2.3.2 Inclusion and exclusion criteria

The inclusion criteria of RCTs were as follows: (1) Participants were patients with non-valvular AF; (2) Included studies specified patient inclusion and exclusion criteria; (3) Interventions were TCM or western drug therapies, with control groups receiving TCM or western drug therapies, placebo, or no intervention. TCM interventions encompassed various proprietary Chinese medicines, hospital-prepared formulations, and self-formulated prescriptions; (4) Study design was RCT.

The exclusion criteria of RCTs were as follows: (1) Duplicate publications; (2) Incomplete literature reports; (3) Non-pharmacological treatments, such as acupuncture, massage, or acupoint patch; (4) Reviews, degree theses, or conference abstracts.

#### 2.3.3 Information extraction

The working group imported all search results into NoteExpress (version 3.8.0.9520) for duplicate removal. Abstracts and full texts were screened by two reviewers (CZ and XW), and any disagreements will be resolved through discussion with a third researcher (BP). The number of articles excluded at each stage was recorded throughout the process. Studies that met the inclusion criteria were assessed using a standardized data extraction form in Excel software. The following information was collected: (1) Study basic information: title, authors, journal, publication year, sample size, number of groups, multicenter status, trial registration status; (2) Subject characteristics: disease name, TCM syndrome pattern, enrollment time period, and age range; (3) Intervention measures: intervention methods, route of administration, treatment duration, dosage and frequency; (4) Outcomes, measurement instruments, and measurement time points. The data extraction process was cross-checked by two researchers (BC and JQ). Any discrepancies were resolved through discussion with a third researcher (JW).

### 2.4 Clinical research information

#### 2.4.1 Physician questionnaire survey

The online questionnaire survey aims to collect outcomes of interest to clinicians, ensuring the representativeness and clinical utility. On the premise of ensuring physicians' privacy security, researchers invited them to provide their name, practice category (TCM practitioner, western medicine practitioner, or integrated medicine practitioner), hospital tier, etc*.*, to support subsequent Delphi questionnaire survey. Physicians participating in the questionnaire should have sufficient geographic representation, drawn from multiple municipalities across the nation, as well as hospitals of varying tiers.

#### 2.4.2 Semi-structured patient interviews

As the subjects of clinical trials, patients’ experiences and concerns are of great importance. Semi-structured interviews were conducted to maintain the research focus while enhancing the completeness and reliability of information collection, thereby ensuring the comprehensiveness of the outcomes collected in this study. All participating patients provided informed consent before the interviews were conducted. During the interviews, medical terminology was avoided in favor of colloquial language. After explaining the study overview and obtaining consent, the interview was initiated with open-ended questions. Discussions then progressed sequentially and in greater depth based on the patients’ responses. Patient interviews were conducted iteratively until no new outcomes were identified. Sample adequacy was therefore considered in relation to the study aim, participant diversity, and the richness of the data obtained, rather than being determined by formal power-based sample size calculation (Malterud et al., 2016).

### 2.5 Data standardization

Unified the coding of collected outcomes for subsequent statistical analysis and traceability. Merged outcomes with different names but identical definitions, including their Chinese and English terms and abbreviations, such as therapeutic efficacy and clinical efficacy, interleukin-6 and IL-6, C-reactive protein and CRP. Retained outcomes with overlapping definitions, such as clinical efficacy and TCM syndrome efficacy, thrombotic event rate and stroke incidence. Deleted duplicate or undefined outcomes. Employed descriptive analysis to conduct frequency statistics on outcomes. For TCM syndromes, patterns, and symptoms mentioned in the involved studies, standardization was achieved by referencing the *Terminology for Clinical Diagnosis and Treatment of Traditional Chinese Medicine Part 2:TCM Syndrome Patterns (GB/T16751.2-2021)* issued by the National Administration of Traditional Chinese Medicine and the National Health Commission (NATCM and NHC, 2020).

### 2.6 Consensus process

#### 2.6.1 Delphi questionnaire survey

This study employed two rounds of Delphi questionnaire surveys. The outcomes included in the first-round questionnaire were derived from the preliminary outcome pool. Separate questionnaires were developed for patients and other stakeholder groups, with the patient version using more accessible language and the other versions using more specialized terminology. The importance of each outcome was assessed using a nine-point Likert scale, with higher scores indicating greater importance. Scores of one to three were considered “not important”, 4–6 as “important but not critical”, and 7–9 as “critical”. Respondents could also select “Unsure” if they were unable to judge an outcome’s importance. As this Delphi survey was conducted to support outcome prioritization and consensus building in COS development rather than statistical inference, the panel size was determined with consideration of stakeholder representation and recruitment feasibility rather than formal statistical power calculation (Nasa et al., 2021). The questionnaire also included open-ended questions, allowing participants to add outcomes they considered important but that were not covered in the questionnaire. The working group reviewed these additional outcomes to determine whether they should be incorporated into the second-round questionnaire. In the second round, measurement instruments and time points were further included for evaluation. For each survey round, the expert authority coefficient, coefficient of variation (*CV*), and Kendall’s coefficient of concordance (*W*) were calculated. A lower CV indicated greater consistency in outcome scoring. Kendall’s W ranges from 0 to one and was tested using the chi-square test, with higher values indicating stronger agreement among participants. The response period for each round was 2 weeks.

#### 2.6.2 Consensus definition

After completing a round of surveys, if at least 70% of participants rated an outcome as “important” (scoring 4–9 points), it was deemed “consensus reached” and proceeded to the second round of surveys or a consensus meeting. If over 70% of respondents deemed an outcome “unimportant” (scoring 1–3 points), it was deemed “consensus rejected” and excluded. The remaining outcomes were collectively discussed by working group members to determine retention. In the consensus meeting, outcomes deemed “important” (scoring 4–9 points) by over 75% of members were considered consensus-reached and incorporated into the COS-TCM-AF. Measurement instruments and measurement time points deemed “important” (scoring 4–9 points) by over 75% of members were recommended. Participants would discuss the remaining outcomes, measurement instruments, and measurement time points where consensus had not been achieved. Decisions regarding their inclusion or exclusion would be based on their importance, practicality, and representativeness. The final COS, recommended measurement instruments, and time points would be determined accordingly.

#### 2.6.3 Consensus meeting

To select the final outcomes for inclusion in the COS-TCM-AF, representatives from stakeholder groups who actively and fully participated in both rounds of the Delphi survey, along with members of the COS development working group, were invited to attend the consensus meeting. The working group initially presented an overview and findings of the study. Participants were subsequently invited to cast anonymous votes through an online Questionnaire Star survey, selecting either “important” or “unimportant” for each outcome, measurement instrument, and measurement time point listed in the consensus meeting inventory. The working group members tallied the statistical results, presented the documentation, and organized the meeting content. The COS-TCM-AF was established in accordance with the consensus result.

## 3 Results

### 3.1 Literature and registered trials

**FIGURE 1:**
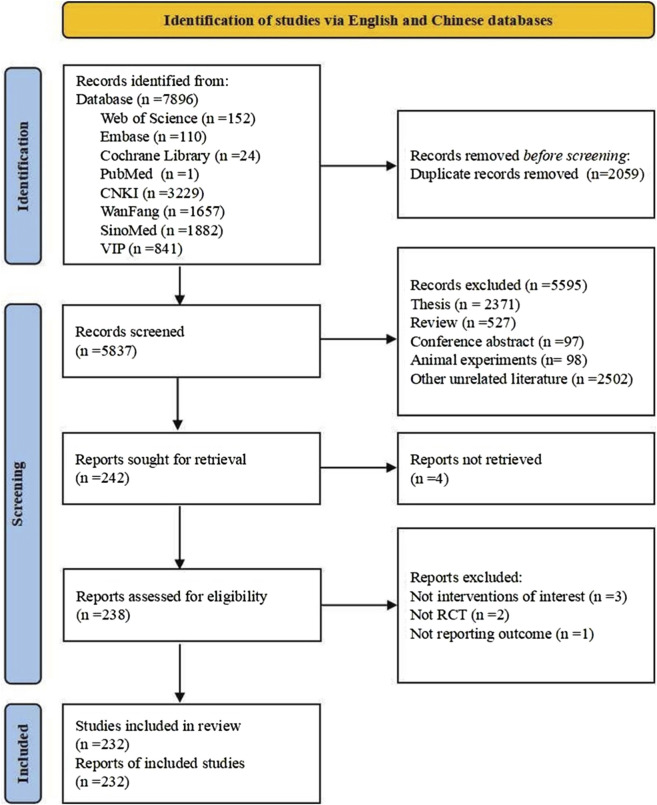
PRISMA flow diagram.

A total of 7,896 literature records were retrieved, with 5,837 remaining after removing duplicates. Initial screening based on titles and abstracts excluded 5,595 records, including theses, conference abstracts, reviews, and animal studies. Further full-text review eliminated 10 studies due to comorbidity, unavailable full texts, non-compliant interventions, non-RCTs, or unreported outcome measures. Finally, 232 RCTs were included in the literature, with a total of 993 outcomes. The literature screening process is shown in Figure 1. Detailed characteristics of the included studies are provided in Supplementary Table S2. After merging outcomes with the same name and removing those erroneously reported, there were 163 outcomes with 977 reporting frequencies. A total of 166 registered trials were collected, with five meeting criteria for inclusion, all from the Chinese Clinical Trial Registry. There were 20 reported outcomes with a reporting frequency of 26 times.

### 3.2 Clinical research

#### 3.2.1 Physician questionnaire survey

A total of 96 questionnaires were collected from clinicians at 63 tertiary hospitals across 22 provinces and municipalities nationwide. Of the participating physicians, 89.58% (86/96) were from cardiovascular departments, 80.21% (77/96) were aged 40 or older, 63.54% (61/96) had over 20 years of professional experience, and 62.50% (60/96) held doctoral degrees. The questionnaire results were aggregated, and the reported outcomes were standardized and statistically organized, yielding a total of 24 outcomes with 1,136 frequencies.

#### 3.2.2 Patient semi-structured interview

23 patients from 12 hospitals across eight provinces and municipalities participated in interviews, 23 completed interview records were collected. Three patients were under the age of 50, while 10 patients were aged between 50 and 70, and another 10 were over 70. By source, 15 were inpatients and eight were outpatients. Among them, 18 had paroxysmal AF, four had persistent AF, and one had permanent AF. The interview results were compiled and 23 outcomes were reported, with a reporting frequency of 173 times.

### 3.3 Preliminary outcome pool

All outcomes were standardized and collated, and reporting frequency statistics were calculated. A total of 182 outcomes (with 2,312 reporting frequencies) were collected and categorized into eight domains: clinical examination, symptoms and signs, safety events, TCM symptoms, quality of life, long-term prognosis, economic evaluation, and patient satisfaction. To enhance feedback efficiency, the steering committee deliberated and decided to exclude outcomes that at least 90% of members agreed on the following criteria: (1) Weak relevance to atrial fibrillation disease; (2) Poor specificity, (3) Lack of clinical value; (4) Overlapping conceptual content. Additionally, outcomes unanimously deemed important but not previously mentioned were supplemented. This resulted in a preliminary outcome pool comprising 36 outcomes across seven domains (Table 1).

**TABLE 1:** Average scores and proportion of seven to nine of outcomes in Delphi survey and consensus meeting.

Outcome domain	Outcomes	Delphi round 1		Delphi round 2		Consensus meeting	
		Average	Proportion of 7–9	Average	Proportion of 7–9	Average	Proportion of 7–9
Clinical examination	Electrocardiogram (ECG) outcomes	7.84	97.65%	7.73	82.86%	7.58	73.68%
	Echocardiogram outcomes/cardiac magnetic resonance (CMR) outcomes	7.51	96.47%	7.34	80.00%	6.89	57.89%
	NT-ProBNP/BNP	7.34	94.19%	7.27	77.14%	7.21	68.42%
	International normalized ratio (INR)	7.51	94.81%	6.83	68.57%	6.37	47.37%
	Soluble suppression of tumorigenicity 2 (sST2)	6.86	92.31%	6.61	61.43%	—	—
	Prothrombin time (PT)	7.18	96.10%	6.43	60.00%	—	—
	Galectin-3 (Gal-3)	6.66	92.21%	6.34	52.86%	—	—
	Interleukin-6 (IL-6)	6.48	91.25%	6.29	47.14%	—	—
Symptoms and signs	Ventricular rate	7.52	96.51%	7.41	80.00%	7.00	63.16%
	AF episode frequency	7.54	95.24%	7.37	72.86%	8.37	94.74%
	AF burden	7.30	95.24%	7.20	71.43%	8.26	89.47%
	AF episode duration	7.25	95.24%	7.17	72.86%	7.74	89.47%
TCM syndrome	Palpitation	7.65	96.47%	7.39	72.86%	7.89	89.47%
	Tcm syndrome efficacy	7.43	96.34%	7.31	72.86%	6.37	36.84%
	Chest tightness	7.03	94.19%	6.91	57.14%	—	—
	Fatigue	7.01	96.51%	6.81	58.57%	—	—
	Shortness of breath	6.94	96.51%	6.60	52.86%	—	—
	Dizziness	6.66	93.02%	6.37	47.14%	—	—
Quality of life	Quality of life assessment	7.75	97.65%	7.51	74.29%	6.95	73.68%
	Depressive state	6.82	94.25%	6.91	67.14%	—	—
	Sleep quality	6.80	95.35%	6.81	62.86%	—	—
	Anxious state	6.90	95.35%	6.91	67.14%	6.16	63.16%
Long-term prognosis	Thromboembolic(TE) event rate	7.83	95.35%	7.73	81.43%	8.05	89.47%
	Incidence of acute heart failure/acute exacerbation of chronic heart failure	7.76	96.55%	7.67	81.43%	7.89	84.21%
	AF recurrence rate	7.68	98.75%	7.24	74.29%	8.16	89.47%
	Rehospitalization rate caused by af episodes	7.49	95.00%	7.04	70.00%	7.42	68.42%
	Emergency department visit rate caused by af episodes	7.39	95.00%	7.00	67.14%	—	—
	AF recurrence time	7.26	92.50%	6.84	61.43%	—	—
	Length of hospital stay	6.59	91.76%	6.61	58.57%	—	—
Safety events	Bleeding event rate	7.40	94.19%	7.34	72.86%	7.26	73.68%
	Renal function	7.07	94.19%	7.06	72.86%	6.26	47.37%
	Liver function	6.81	92.94%	6.93	68.57%	—	—
	Incidence of idiopathic pulmonary fibrosis	6.60	89.16%	6.51	55.71%	—	—
	Incidence of thyroid dysfunction	6.54	90.36%	6.40	48.57%	—	—
	Incidence of photosensitivity reaction	5.92	83.33%	5.70	37.14%	—	—
Economic evaluation	Treatment cost	6.94	94.19%	6.93	67.14%	—	—

### 3.4 Delphi survey

#### 3.4.1 Round one of the delphi survey

100 questionnaires were distributed in the first round, with the data collection period spanning from December 15 to 31 December 2024. A total of 90 completed questionnaires were retrieved. After excluding three invalid or duplicate responses, the expert response rate was 87.00% (87/100). Among the 87 valid responses, 13 were from patients and their caregivers, 74 were from physicians, clinical research methodologists, journal editors, pharmaceutical representatives and others (Table 2). Except for patients and their caregivers, 59.46% (44/74) hold senior professional titles, and 45.95% (34/74) have over 20 years of work experience. On completion of the first round, all 36 outcomes met the predefined consensus criteria. No outcomes were excluded. The majority of outcomes from the first round displayed low coefficient of variation (W = 0.138, P < 0.001), suggesting strong consistency yet poor coordination among expert scores (Table 1).

**TABLE 2:** Demographic characteristics of participants of Delphi survey and consensus meeting.

Characteristics	Delphi round 1	Delphi round 2	Consensus meeting
Stakeholder group	(n = 87)	(n = 70)	(n = 19)
Cardiologist	58 (66.67%)	43 (61.43%)	8 (42.11%)
TCM physician	37/58 (63.79%)	27/43 (62.79%)	4/8 (50.00%)
Western physician	10/58 (17.24%)	9/43 (20.93%)	2/8 (25.00%)
Integrative medicine physician	11/58 (18.97%)	7/43 (16.28%)	2/8 (25.00%)
Clinical research methodologist	7 (8.05%)	7 (10.00%)	4 (21.05%)
Journal editor	3 (3.45%)	3 (4.29%)	2 (10.53%)
Pharmaceutical representative	4 (4.60%)	4 (5.71%)	3 (15.79%)
Other (Pharmacist, Nurse)	2 (2.30%)	2 (2.86%)	0
Patients and caregivers	13 (14.94%)	11 (15.71%)	2 (10.53%)
Professional qualification	(n = 74)	(n = 59)	(n = 17)
Senior	44/74 (59.46%)	35/59 (59.32%)	10/17 (58.82%)
Intermediate	28/74 (37.84%)	23/59 (38.98%)	4/17 (23.53%)
Junior	2/74 (2.70%)	1/59 (1.69%)	3/17 (17.65%)
Years of work experience	(n = 74)	(n = 59)	(n = 17)
>20 years	34/74 (45.95%)	29/59 (49.15%)	7/17 (41.18%)
10–20 years	32/74 (43.24%)	26/59 (44.07%)	7/17 (41.18%)
<10 years	8/74 (10.81%)	4/59 (6.78%)	3/17 (17.65%)
Region	(n = 20)	(n = 17)	(n = 7)
Beijing	22/87 (25.29%)	15/70 (21.43%)	3/19 (15.79%)
Tianjin	12/87 (13.79%)	11/70 (15.71%)	5/19 (26.32%)
Shandong	11/87 (12.64%)	10/70 (14.29%)	2/19 (10.53%)
Xinjiang	8/87 (9.20%)	7/70 (10.00%)	—
Hebei	5/87 (5.75%)	6/70 (8.57%)	2/19 (10.53%)
Shaanxi	4/87 (4.60%)	4/70 (5.71%)	2/19 (10.53%)
Shanghai	4/87 (4.60%)	4/70 (5.71%)	—
Henan	3/87 (3.45%)	1/70 (1.43%)	4/19 (21.05%)
Jilin	3/87 (3.45%)	2/70 (2.86%)	—
Sichuan	3/87 (3.45%)	3/70 (4.29%)	1/19 (5.26%)
Fujian	2/87 (2.30%)	1/70 (1.43%)	—
Guangdong	2/87 (2.30%)	—	—
Gansu	1/87 (1.15%)	1/70 (1.43%)	—
Guangxi	1/87 (1.15%)	1/70 (1.43%)	—
Guizhou	1/87 (1.15%)	—	—
Heilongjiang	1/87 (1.15%)	—	—
Hunan	1/87 (1.15%)	1/70 (1.43%)	—
Jiangsu	1/87 (1.15%)	1/70 (1.43%)	—
Jiangxi	1/87 (1.15%)	1/70 (1.43%)	—
Zhejiang	1/87 (1.15%)	1/70 (1.43%)	—

#### 3.4.2 New entries

An open-ended question was involved at the end of the first-round questionnaire to solicit additional important outcomes from respondents. Among the first-round responses, two participants proposed outcomes overlapped with the existing ones: number of AF episodes and ventricular rate, so no new outcomes were added. Based on literature data extraction and clinical survey results, the second-round Delphi questionnaire supplemented measurement instruments and time point for certain outcomes. The measurement instruments encompassed ECG monitoring devices, cardiac imaging equipment, symptom and efficiency assessment instruments, quality of life assessment scale, sleep quality scale, depression scale, and anxiety scale. Additionally, the time point for measuring recurrence in AF patients after various treatment modalities was included.

#### 3.4.3 Round two of the delphi survey

**FIGURE 2:**
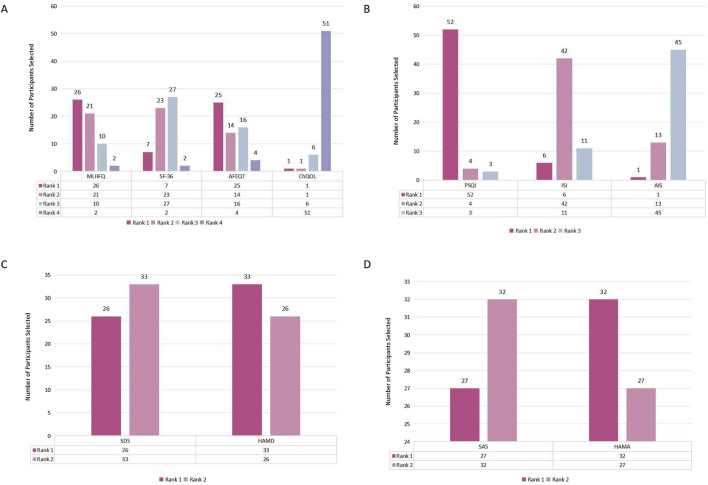
**(A)** Importance ranking of quality-of-life scales; **(B)** Importance ranking of sleep-quality scales; **(C)** Importance ranking of depression assessment scales; **(D)** Importance ranking of anxiety assessment scales.

The second-round Delphi survey was conducted from January 10 to 25 January 2025. The questionnaires were distributed to the 87 participants from the first round, with 70 completed questionnaires returned (Table 2). The expert response rate for the second-round Delphi survey was 80.46% (70/87). Participants involved 59 experts and 11 patients or caregivers. Most outcomes exhibited low coefficient of variation (*W = 0.122, P < 0.001*), indicating consistent scoring but low coordination. A total of 16 outcomes, considered important by at least 70% of all stakeholder groups, were retained for the consensus meeting. Two outcomes with no consensus were excluded. The remaining outcomes were discussed by the working group to determine retention, ultimately adding INR and anxiety status. All 59 experts participated in both the measurement instrument importance ranking and evaluation (Table 3, 4). The measurement instruments importance ranking results are showed in Figure 2. Since the two outcomes of sleep quality and depressive state have been excluded, the corresponding sleep quality scales and depressive scales are no longer recommended as measurement instruments and therefore will not be included in the consensus meeting list.

**TABLE 3:** Average scores and proportion of seven to nine of measurement instruments in Delphi survey and consensus meeting.

Measure domain	Measurement instrument	Round 2 of Delphi survey		Consensus meeting	
		Average	Proportion of 7–9	Average	Proportion of 7–9
ECG monitoring device	Holter monitoring	7.21	67.14%	8.05	89.47%
	Long-term holter monitoring	6.89	60.00%	7.53	78.95%
	Implantable cardiac monitorb (ICM)	5.99	38.57%	—	—
	Smart wearable devices	5.76	37.14%	—	—
Cardiac imaging equipment	Echocardiogram	7.51	80.00%	6.47	36.84%
	Cardiac MR	6.73	57.14%	6.26	42.11%
Symptom and efficiency assessment instrument	EHRA classification (for patients without HF) or NYHA classification (for patients with HF)	7.47	81.43%	7.68	78.95%
	TCM symptom score	7.01	68.57%	7.63	78.95%

**TABLE 4:** Average scores and proportion of seven to nine of measurement time points in Delphi survey and consensus meeting.

Patient population	Measurement time point	Round 2 of Delphi survey		Consensus meeting	
		Average	Proportion of 7–9	Average	Proportion of 7–9
AF patients after electrical cardioversion or pharmacologic cardioversion	Within 3 months	7.37	75.71%	7.74	89.47%
	3–12 months	6.97	62.86%	—	—
	Over 12 months	6.54	57.14%	—	—
AF patients after radiofrequency ablation	3–12 months	7.47	72.86%	7.84	89.47%
	Over 12 months	7.11	71.43%	6.89	47.37%

### 3.5 Consensus meeting

The consensus meeting was convened on 5 March 2025, with 21 participants invited and 19 attending (Table 2). A total of 19 questionnaires were distributed and collected during the meeting with 100% response rate. The meeting conducted importance evaluations for 18 outcomes, measurement instruments and time points (Table1; Table 3, 4). Based on predefined consensus criteria, recommendations were established for the COS-TCM-AF, measurement instruments, and measurement time points. Based on the results of the consensus meeting and expert discussions, a total of seven outcomes across three outcome domains were ultimately included in this COS, along with recommendations for measurement instruments and timing points (Table 5). For electrocardiographic monitoring, Holter monitoring or long-term Holter monitoring is recommended. For symptom and efficacy assessment instruments, TCM symptom score or the EHRA (European Heart Rhythm Association) classification (for patients without HF) or NYHA (New York Heart Association) classification (for patients with HF) are suggested. AF patients who undergo electrical or pharmacologic cardioversion should be monitored for AF recurrence within 3 months post-procedure. Those who undergo radiofrequency ablation should have their monitoring conducted between three and 12 months post-procedure. All experts unanimously recommended using the Minnesota Longitudinal Heart Failure Questionnaire (MLHFQ) for AF patients with HF and the Atrial Fibrillation-Specific Quality of Life Questionnaire (AFEQT) for those without HF to assess quality of life. Regarding anxiety assessment, 73.68% (14/19) respondents recommended the Hamilton Anxiety Rating Scale (HAMA), while 26.32% (5/19) recommended the Self-Rating Anxiety Scale (SAS). However, since neither quality of life nor anxiety status were included as recommended outcomes in COS-TCM-AF, this study does not recommend relevant instruments.

**TABLE 5:** The final COS for AF in clinical trials of TCM.

Outcome domain	Outcomes
Symptoms and signs	AF episode frequency*
	AF episode duration*
	AF burden
TCM symptom	Palpitation
Long-term prognosis	TE event rate
	AF recurrence rate
	Incidence of acute HF/acute exacerbation of chronic HF

* For patients with paroxysmal AF, only.

## 4 Discussion

COS as the most representative collection of outcomes reflecting treatment efficacy, can measure patient health status and disease improvement (Moller, 2019; Lampro and George, 2022). This study systematically collected outcomes from literature, registered trials, clinicians, and patients through online retrieval and clinical surveys. During the outcome collection and organization process, numerous issues were identified in the original studies, including overlapping definitions of outcomes, insufficient attention to TCM outcomes, diverse measurement instrument selections, ambiguous measurement time points, and limited reporting of endpoint outcomes. The seven core outcomes encompass critical aspects, including AF episodes, patient symptoms, complication risks, and long-term prognosis. These are of significant importance for evaluating the efficacy and safety of TCM treatment for AF, providing key reference criteria for clinical practice and research. Although quality of life was not ultimately included in the final core outcome set, this should not be interpreted as indicating that it is unimportant in AF research. Rather, its exclusion reflects the result of the predefined consensus process used in this study. Given that AF affects not only rhythm-related outcomes and complication risk but also symptom perception and patient-reported benefit, quality of life remains a highly relevant patient-centered outcome. Recent evidence has shown that treatment-related factors may influence perceived quality of life and symptom relief after atrial fibrillation treatment (Matteucci et al., 2025). Therefore, future clinical trials of TCM for AF may consider quality of life as an important supplementary endpoint.

Although thromboembolic event rate was included as a final core outcome, improvement in rhythm-related outcomes such as AF recurrence or AF burden does not necessarily indicate elimination of residual thromboembolic risk. In clinical interpretation, thromboembolic outcomes should be considered together with the patient’s CHA_2_DS_2_-VASc profile, anticoagulation status, and duration of follow-up. Particularly after AF ablation, rhythm improvement alone should not be interpreted as sufficient justification for de-escalation of stroke prevention strategies. Recent evidence on anticoagulation after AF ablation and non-anticoagulation approaches to stroke prevention further supports the importance of interpreting thromboembolic outcomes within a broader and more clinically realistic framework (Matteucci et al., 2026; Sgarra et al., 2025).

Throughout this study, we strictly adhered to the series of COS development methods and standards established by the COMET Working Group, ensuring the standardization and scientific rigor of the research process (Kirkham et al., 2019; Kirkham et al., 2017; Williamson et al., 2017; Kirkham et al., 2016). In this methodological framework, the prioritization of outcomes is based on structured consensus among relevant stakeholders rather than regression-based statistical modeling. Therefore, although no inferential statistical modeling was applied, the approach used in this study remains consistent with current methodological recommendations for core outcome set development.

However, there were some limitations of the research. First, the Delphi survey exhibited an imbalance in stakeholder representation. While no fixed standard exists for the number of participants per stakeholder group in Delphi questionnaires, COS-STAD identifies COS users, healthcare professionals, and patients as indispensable participants in the Delphi process (Kirkham et al., 2017). Although this study encompassed these stakeholder groups, their distribution was significantly uneven. For instance, in the second round of Delphi surveys, the number of clinicians far exceeded that of patients and COS users. Such imbalance may potentially influence the study results. Future research should carefully consider the numerical representation of each group to ensure that the outcome measures fully and reliably reflect the perspectives and demands of all stakeholders. Second, the Delphi survey lacked an effective feedback mechanism. In the second round, we distributed individualized questionnaires to each first-round participant, requesting explanations for significant score changes. However, due to technical limitations, participants' previous scores were unmasked. This may have led some participants to maintain their original scores, even if they had developed new insights into outcome importance, to avoid the hassle of explaining the changes. The tendency for participants' scores to align across rounds hindered the questionnaire’s ability to accurately reflect dynamic shifts in expert opinions, failing to capture adjustments in participants' perceptions of outcome importance during iterative interactions. To prevent such issues in future studies, questionnaire design should be optimized by effectively masking participants' previous scores and streamlining the process for explaining score changes to enhance feedback efficiency.

The incorporation of this COS into the clinical research design framework for TCM treatment of AF holds significant value for enhancing study quality (Lampro and George, 2022). COS application effectively circumvents the limitations of traditional clinical trials, which often involve numerous but non-targeted outcomes. By focusing on the most clinically meaningful outcome measures, reducing the measurement and analysis of unnecessary outcomes, optimizing the allocation of research resources, and enhancing the reproducibility and scientific credibility of research results (Zhang Peng, 2025; Xing Dongmei, 2014; Chiarotto et al., 2017). It is recommended that researchers explicitly incorporate COS during the trial protocol design and prioritize the presentation of core outcome measurements in research reports. This approach lays the foundation for establishing an evidence-based medicine system for TCM treatment of AF (Blackwood et al., 2019).

## 5 Conclusion

This COS identifies seven core outcomes to be included, encompassing AF episode frequency, AF episode duration, AF burden, TCM symptoms—palpitations, thromboembolic (TE) event rate, AF recurrence rate, and acute HF/acute exacerbation of chronic HF incidence. This COS comprehensively covers dimensions including AF event occurrence, patient clinical symptoms, complication risks, and long-term prognosis. It also provides recommendations on measurement instruments selection and timing points. Implementation of this COS will facilitate comparison of results across similar studies and serve as a reference for outcome selection and evaluation.

## Data Availability

The original contributions presented in the study are included in the article/Supplementary Material, further inquiries can be directed to the corresponding authors.
