# Supplementary material for: Development of a core outcome set for traditional Chinese medicine for atrial fibrillation
Source: Front Pharmacol. 2026 May 7;17:1731405. doi: 10.3389/fphar.2026.1731405 (PMC13216717; doi:10.3389/fphar.2026.1731405)
Supplement: Supplementary file 1 [file DataSheet2.docx]

Supplementary table 2. Basic information of included studies

| No. | Year | Authors | No. of patients (intervention/control) | Classification | TCM syndrome pattern | Intervention | | Treatment duration (days) |
| --- | --- | --- | --- | --- | --- | --- | --- | --- |
|  |  |  |  |  |  | Intervention group | Control group |  |
| 1 | 1990 | Hu Xiu Zhu et al. | 20/14 | CAF | NK | self-designed cardioversion decoction + Amiodarone | Amiodarone | 28 |
| 2 | 1997 | Yang Fa Rong et al. | 32/28 | AF with RVR | NK | self-designed cardioversion decoction + Shenmai Injection | verapamil | 30 |
| 3 | 2003 | Wang Fu Ji et al. | 62/48 | PAF | NK | Wenxin Keli | propafenone | 28 |
| 4 | 2003 | Wei Pei Feng et al. | 30/30 | PAF | dual deficiency of qi and yin + heart-blood stasis obstruction | Wenxin Keli | propafenone | 28 |
| 5 | 2003 | Hong Yan Zhang et al. | 26/26 | PAF | NK | Shenqi Tablets | vitamin C tablets | 360 |
| 6 | 2003 | Xing Tao et al. | 33/27 | PeAF | spleen-qi deficiency + dual deficiency of qi and yin + qi deficiency and blood stasis + heart-lung qi deficiency | self-designed cardioversion decoction | Amiodarone | 56 |
| 7 | 2004 | Yang Zhi Xin et al. | 20/20 | PeAF | NK | Xinanning nasal drops | Amiodarone | 28 |
| 8 | 2005 | Zhang Jin Feng et al. | 30/30/30 | PAF | NK | Wenxin Keli | 1 propafenone, 2 moracizine | 180 |
| 9 | 2005 | Li Jun et al. | 47/43 | NK | NK | Shexiang Baoxin Pills + Amiodarone + perindopril | Amiodarone + perindopril | 28 |
| 10 | 2005 | Wang Shou Fu et al. | 56/54 | NK | NK | Fumai Huoxue Decoction | Amiodarone + aspirin | 60 |
| 11 | 2005 | Zhao Wei et al. | 106/62 | NK | NK | Puerarin Injection + Shenmai Injection + propafenone | propafenone | 14 |
| 12 | 2006 | Wang Zhao Wei et al. | 37/39 | PeAF | NK | Wenxin Keli + digoxin | digoxin | NK |
| 13 | 2006 | Hu Mei Qin et al. | 50/50 | PAF | NK | Wenxin Keli | propafenone | 28 |
| 14 | 2006 | Zhao Ze Hong et al. | 152/109 | NK | NK | Xianling Powder + Amiodarone | Amiodarone | 28 |
| 15 | 2006 | Wang Shi Han et al. | 36/32 | PAF | NK | Huxin Capsules + Amiodarone | Amiodarone | 30 |
| 16 | 2006 | Wang Min et al. | 112/112 | NK | NK | Wenxin Keli + Amiodarone | Amiodarone | 180 |
| 17 | 2007 | Luo Fang et al. | 36/34 | AF with RVR | NK | self-designed Yangxin Dingji Decoction | deslanoside injection | 21 |
| 18 | 2007 | Xu Jin Feng et al. | 38/20 | NK | NK | Modified Zhigancao Decoction | Amiodarone | 30 |
| 19 | 2007 | Chen Shou Hong et al. | 44/44 | PermAF | NK | self-designed Yiqi Huoxue Formula + conventional treatment | conventional treatment | 30 |
| 20 | 2007 | Tang Ming Chun et al. | 30/28 | PAF | NK | Shenmai Injection + self-designed TCM formula | verapamil | 30 |
| 21 | 2007 | Zheng Yan Sheng et al. | 21/21 | PAF | NK | Wenxin Keli | digoxin | 28 |
| 22 | 2007 | Li Jian Guang et al. | 60/64 | AF with RVR | NK | Shengmai Injection + Amiodarone + aspirin | Amiodarone + aspirin | 21 |
| 23 | 2007 | Han Hong Yan et al. | 60/60 | PAF | NK | Shensong Yangxin Capsules + digoxin + metoprolol | digoxin + metoprolol | 90 |
| 24 | 2007 | Wu Peng Tao et al. | 58/55/54 | PAF | NK | Shensong Yangxin Capsules + metoprolol | 1 metoprolol, 2 Shensong Yangxin Capsules | 90 |
| 25 | 2008 | Liu Bing et al. | 44/44 | AF with RVR | NK | Wenxin Keli + metoprolol | metoprolol | 28 |
| 26 | 2008 | Zhang Shu Lin et al. | 42/42 | PermAF | NK | Shensong Yangxin Capsules + conventional treatment | conventional treatment | 180 |
| 27 | 2008 | Meng Zi Min et al. | 40/38 | PermAF | NK | Wenxin Keli + conventional treatment | conventional treatment | 90 |
| 28 | 2008 | Ma Shu Nuo et al. | 47/48 | NK | NK | Wenxin Keli + Amiodarone | Amiodarone | 90 |
| 29 | 2008 | Qu Yun Zhong et al. | 40/36 | PAF | NK | Wenxin Keli + propafenone | propafenone | 28 |
| 30 | 2008 | Wu Peng Tao et al. | 57/61/59 | PAF | NK | Wenxin Keli + bisoprolol | 1 bisoprolol, 2 Wenxin Keli | NK |
| 31 | 2008 | Yang Da Nan et al. | 51/44 | PAF | NK | Xinkeshu Tablets | aspirin | 180 |
| 32 | 2008 | Yan Fang et al. | 41/64 | NK | NK | Dayu Decoction + warfarin | warfarin | 360 |
| 33 | 2008 | Li Hai Tao et al. | 26/20 | acute AF | NK | Shensong Yangxin Capsules + Amiodarone | Amiodarone | 168 |
| 34 | 2008 | Zhang Jin Mei et al. | 30/31 | AF with RVR | NK | Wenxin Keli + metoprolol | metoprolol | 28 |
| 35 | 2008 | Chen Zhe et al. | 26/25 | PermAF | NK | Wenxin Keli + metoprolol | metoprolol | 180 |
| 36 | 2008 | Wu Jun Lan et al. | 30/30/30 | PAF | NK | Wenxin Keli + captopril | 1 captopril, 2 Wenxin Keli | 180 |
| 37 | 2009 | Sun Fu Jun et al. | 82/68 | PeAF | NK | Wenxin Formula | Amiodarone | 28 |
| 38 | 2009 | Hu Zhi Geng et al. | 40/38 | PAF | NK | cardioversion decoction + Amiodarone | Amiodarone | 30 |
| 39 | 2009 | Wang Chun Tong et al. | 63/62 | PAF | NK | self-designed Yangxin Huoxue Formula + Amiodarone | Amiodarone | 360 |
| 40 | 2009 | Lu Yu Hong et al. | 60/46 | CAF | NK | Tongxinluo Capsules | warfarin | NK |
| 41 | 2009 | Ji Jian Jiang et al. | 30/60 | PAF | NK | Shensong Yangxin Capsules + propafenone | propafenone | 56 |
| 42 | 2009 | Yang Hu et al. | 42/26 | PAF | NK | self-designed Dingxin Fuli Decoction | Amiodarone | 30 |
| 43 | 2009 | Zhao Wen Xue et al. | 76/62 | PAF | NK | self-designed Yangxue Fumai Beverage | propafenone | 28 |
| 44 | 2009 | He Yu Juan et al. | 39/37/42 | PAF | NK | Wenxin Keli + Amiodarone | 1 Amiodarone, 2 Wenxin Keli | 180 |
| 45 | 2009 | Song Guang Yao et al. | 35/40 | PeAF | NK | Shensong Yangxin Capsules | digoxin + metoprolol | 56 |
| 46 | 2009 | Xing Jun et al. | 18/22 | PAF | NK | Wenxin Keli + Amiodarone | Amiodarone | NK |
| 47 | 2009 | Yang Nan Ying et al. | 33/32 | PAF | NK | Wenxin Keli + Amiodarone | Amiodarone | NK |
| 48 | 2010 | Zhang Xiu Feng et al. | 50/50 | PermAF | NK | Shensong Yangxin Capsules + digoxin + metoprolol + nifedipine | digoxin + metoprolol + nifedipine | 180 |
| 49 | 2010 | Sheng Hong Ping et al. | 30/30 | PAF | NK | Shensong Yangxin Capsules + conventional treatment | conventional treatment | NK |
| 50 | 2010 | Gao Song et al. | 32/32 | PAF | NK | Wenxin Keli | Amiodarone | NK |
| 51 | 2010 | Liu Ying Hua et al. | 40/40 | PAF | NK | Shensong Yangxin Capsules + Amiodarone | Amiodarone | 720 |
| 52 | 2010 | Lu Nuo et al. | 60/60 | PAF | NK | Wenxin Keli + Amiodarone | Amiodarone | 360 |
| 53 | 2010 | Zhang Jin Hong et al. | 30/30/30 | PAF | NK | Wenxin Keli | 1 propafenone, 2 moracizine | NK |
| 54 | 2010 | Li Hua De et al. | 42/42 | NK | NK | Wenxin Keli + Amiodarone + warfarin | Amiodarone + warfarin | 30 |
| 55 | 2010 | Hui Jin Kai et al. | 34/30 | PeAF | NK | Yangxinshi Tablets | Amiodarone | 84 |
| 56 | 2010 | Han Yan Hua et al. | 50/50 | PAF | NK | Shensong Yangxin Capsules + conventional treatment | conventional treatment | 180 |
| 57 | 2010 | Li Fei Zhou et al. | 28/18 | CAF | NK | Modified Shengmai Decoction + Amiodarone | Amiodarone | 28 |
| 58 | 2010 | Zhuang Xiao Mei et al. | 30/30 | PAF | NK | Shensong Yangxin Capsules | conventional treatment | 90 |
| 59 | 2010 | Fang Fang et al. | 60/60 | PAF | NK | TCM decoction for replenishing qi, activating blood, and calming the mind | conventional treatment | 30 |
| 60 | 2010 | Chen Xi Dong et al. | 40/40 | PAF | NK | Shensong Yangxin Capsules + metoprolol | metoprolol | 28 |
| 61 | 2010 | Li Shi Feng et al. | 33/32 | PAF | NK | Wenxin Keli + Amiodarone | Amiodarone | NK |
| 62 | 2010 | Shao Ji Rui et al. | 31/32 | NK | NK | Shensong Yangxin Capsules | Amiodarone | 30 |
| 63 | 2010 | Xu Ying et al. | 56/56 | PAF | NK | Wenxin Keli + fluvastatin | fluvastatin | 180 |
| 64 | 2010 | Mo Su Qin et al. | 47/47 | PAF | NK | Wenxin Keli + Amiodarone | Amiodarone | 360 |
| 65 | 2010 | Gao Hong Mei et al. | 30/30 | PeAF | NK | Yiqi Huoxue Formula + aspirin | aspirin | 14 |
| 66 | 2010 | Yu Zhong Wei et al. | 58/54 | PAF | qi deficiency and blood stasis | Shensong Yangxin Capsules + Amiodarone | Amiodarone | 90 |
| 67 | 2011 | Shang Lian Yong et al. | 38/38 | NK | NK | cardioversion decoction + Amiodarone + warfarin + low-molecular-weight heparin | Amiodarone + warfarin + low-molecular-weight heparin | NK |
| 68 | 2011 | Yu Yang et al. | 40/40 | CAF | NK | Zhigancao Decoction | metoprolol | NK |
| 69 | 2011 | Chen Zhi Jie et al. | 36/30 | NK | heart-blood stasis obstruction | self-designed Yiqi Huoxue Formula + metoprolol + aspirin | metoprolol + aspirin | 14 |
| 70 | 2011 | Ma Min Kai et al. | 31/30 | PAF | NK | compound concentrated TCM solution | Amiodarone | 60 |
| 71 | 2011 | Zhang Li Min et al. | 48/48 | PAF | NK | Shensong Yangxin Capsules + Amiodarone | Amiodarone | 30 |
| 72 | 2011 | Huang Xia et al. | 44/44 | PAF | NK | Wenxin Keli + Amiodarone | Amiodarone | 365 |
| 73 | 2011 | Liu Hua et al. | 55/53 | PAF | NK | Shensong Yangxin Capsules | conventional treatment | NK |
| 74 | 2011 | Cao Xi Gui et al. | 54/50 | PAF | NK | Wenxin Keli | Amiodarone | 84 |
| 75 | 2011 | Zhang Yan Hong et al. | 40/40 | AF with RVR | NK | Shenqi Fuli Decoction | Amiodarone | 60 |
| 76 | 2011 | Wang Bin et al. | 48/48 | PAF | NK | Shensong Yangxin Capsules + Amiodarone | Amiodarone | 720 |
| 77 | 2011 | Guo Hong Wei et al. | 32/32 | PeAF | phlegm-stasis obstruction + qi deficiency and blood stasis + dual deficiency of qi and yin | Yixin Fushuai Decoction + Amiodarone | Amiodarone | 28 |
| 78 | 2011 | Pang Ling Pin et al. | 30/30 | NK | NK | Zhenzhu Ningxin Decoction + Amiodarone + warfarin | Amiodarone + warfarin | 30 |
| 79 | 2011 | Liu Qiang et al. | 93/87 | NK | dual deficiency of qi and yin + insufficiency of heart yang + internal disturbance of phlegm-heat + heart-blood stasis obstruction | Wenxin Keli for dual deficiency of qi and yin; Yixinshu Capsules for insufficiency of heart yang (qi); He Dan Tablets for disturbance of the heart by phlegm-heat (and stasis); Tongxinluo Capsules for heart-vessel stasis obstruction | conventional treatment | 56 |
| 80 | 2011 | Yuan Ren Xian et al. | 60/60 | PAF | NK | Shensong Yangxin Capsules | Amiodarone | 180 |
| 81 | 2011 | Xiang Xue Jun et al. | 30/30/30 | PAF | NK | Wenxin Keli + Amiodarone | 1 Amiodarone, 2 Wenxin Keli | 56 |
| 82 | 2011 | Zhang Jin Zhu et al. | 45/45 | PAF | NK | Wenxin Keli | Amiodarone | 56 |
| 83 | 2011 | Li Qin et al. | 71/68 | NK | NK | Wenxin Keli | Amiodarone | 56 |
| 84 | 2011 | Wang Yan Li et al. | 33/34 | PermAF | NK | Wenxin Keli + bisoprolol | bisoprolol | 56 |
| 85 | 2011 | Yan Yong Jin et al. | 40/40 | PAF | NK | Wenxin Keli + Amiodarone | Amiodarone | 360 |
| 86 | 2011 | Xie Di Zhong et al. | 30/30 | PAF | NK | Wenxin Keli + metoprolol | metoprolol | 84 |
| 87 | 2011 | Zhang Yu Rong et al. | 30/30 | CAF | NK | Wenxin Keli | Amiodarone | 28 |
| 88 | 2011 | Zhou Qing Guo et al. | 42/41 | NK | NK | Shensong Yangxin Capsules + Amiodarone | Amiodarone | 180 |
| 89 | 2012 | Zhang Cheng Jun et al. | 38/38 | PAF | NK | Wenxin Keli + Amiodarone | Amiodarone | 180 |
| 90 | 2012 | Jin Yi Wu et al. | 24/22/21 | PAF | NK | Shensong Yangxin Capsules + metoprolol | 1 metoprolol, 2 Shensong Yangxin Capsules | 90 |
| 91 | 2012 | Men Rui et al. | 42/42 | PAF | NK | Shensong Yangxin Capsules | Amiodarone | 90 |
| 92 | 2012 | Wang Li Nuo | 21/20 | PAF | NK | Wenxin Keli + metoprolol | metoprolol | 28 |
| 93 | 2012 | Zhang Zhan Wen et al. | 52/50 | PAF | NK | Wenxin Keli + bisoprolol | bisoprolol | NK |
| 94 | 2012 | Lu Jian Zhong et al. | 58/56 | PAF | NK | Shensong Yangxin Capsules + aspirin + simvastatin | aspirin + simvastatin | 180 |
| 95 | 2012 | Li Xiu Yun et al. | 60/56 | NK | NK | Shensong Yangxin Capsules + Amiodarone | Amiodarone | 28 |
| 96 | 2012 | Wang Ai Hu et al. | 106/96/106 | PAF | NK | Shensong Yangxin Capsules + propafenone | 1 propafenone + placebo, 2 Shensong Yangxin Capsules + placebo | NK |
| 97 | 2012 | Su Qi Ying et al. | 33/30 | PermAF | NK | Shengmai Injection + conventional treatment | conventional treatment | 28 |
| 98 | 2012 | Gao Bo et al. | 30/30 | PAF | NK | Wenxin Keli | conventional treatment | NK |
| 99 | 2012 | He Xiao Li et al. | 20/20 | PermAF | insufficiency of heart yang | Wenxinlü Mixture + conventional treatment | conventional treatment | NK |
| 100 | 2012 | Tang Yun Ke et al. | 50/50 | PAF | NK | Shensong Yangxin Capsules + conventional treatment | conventional treatment | 56 |
| 101 | 2012 | Jiang Ying Ming et al. | 89/89 | NK | NK | Wenxin Keli | Amiodarone | 28 |
| 102 | 2012 | Xu Hua Qing et al. | 50/48 | NK | NK | Wenxin Keli + metoprolol | metoprolol | NK |
| 103 | 2012 | Zhang Shuang Quan et al. | 38/38 | NK | NK | Jiuchan Fuli Decoction + amiodarone tablets + aspirin | Amiodarone + aspirin | 28 |
| 104 | 2012 | Chen Hui et al. | 30/30 | PeAF | internal disturbance of phlegm-heat | Qingre Fumai Decoction + conventional treatment | conventional treatment | 21 |
| 105 | 2012 | Zhao Hong Xia et al. | 46/40 | AF with RVR | NK | Shensong Yangxin Capsules + Amiodarone | Amiodarone | 28 |
| 106 | 2013 | Yang Su Xia et al. | 50/50 | NK | NK | Yiqi Huoxue Qufeng Decoction | Amiodarone | 30 |
| 107 | 2013 | Zhu Hao et al. | 51/51 | AF with RVR | phlegm-stasis obstruction | Xuandao Xiefei Beverage + digoxin + benazepril + aspirin + isosorbide mononitrate | digoxin + benazepril + aspirin + isosorbide mononitrate | 7 |
| 108 | 2013 | Shao Jia Kui et al. | 40/40 | PAF | NK | Shensong Yangxin Capsules + rosuvastatin | rosuvastatin | 56 |
| 109 | 2013 | Bao En Yi et al. | 38/38 | AF with RVR | NK | Wenxin Keli + metoprolol | metoprolol | 84 |
| 110 | 2013 | Zheng Qi Hui et al. | 50/50 | PAF | NK | Wenxin Keli + Amiodarone | Amiodarone | 270 |
| 111 | 2013 | Xu Hong Shu et al. | 20/14 | PermAF | NK | Shensong Yangxin Capsules + Amiodarone + aspirin | Amiodarone + aspirin | NK |
| 112 | 2013 | Chang Jia et al. | 23/22 | PAF | NK | Wenxin Keli + Amiodarone | Amiodarone | NK |
| 113 | 2013 | Huang Jian Fen et al. | 55/55 | NK | NK | Wenxin Keli + propafenone injection | propafenone injection | NK |
| 114 | 2013 | Du Chang Li et al. | 46/46 | PeAF | NK | Shensong Yangxin Capsules + aspirin | aspirin | 56 |
| 115 | 2013 | Wang Shang Kun et al. | 43/43 | PAF, PeAF, PermAF | NK | Ginkgo leaf tablets + warfarin | warfarin | 28 |
| 116 | 2013 | Tao Yuan et al. | 28/28 | NK | NK | Wenxin Keli + propafenone | propafenone | 28 |
| 117 | 2013 | Sun Hong Wei et al. | 36/36 | PAF | NK | Shensong Yangxin Capsules + conventional treatment | conventional treatment | 90 |
| 118 | 2014 | Wang Xing Li et al. | 42/40 | PAF | NK | Wenxin Keli + Amiodarone | Amiodarone | 28 |
| 119 | 2014 | Li Li et al. | 56/54 | PAF | NK | Shensong Yangxin Capsules + bisoprolol | bisoprolol | 56 |
| 120 | 2014 | Xu Shu et al. | 28/28 | PAF | NK | Shensong Yangxin Capsules + Amiodarone | Amiodarone | 730 |
| 121 | 2014 | Zheng Min et al. | 30/30 | CAF | NK | Wenxin Keli + Amiodarone | Amiodarone | 28 |
| 122 | 2014 | Li Xin Ling et al. | 42/42 | PAF | NK | Wenxin Keli + Amiodarone | Amiodarone | 180 |
| 123 | 2014 | Zhao Bao Yuan et al. | 41/41 | PAF | NK | Shensong Yangxin Capsules + Amiodarone | Amiodarone | NK |
| 124 | 2014 | Su Jin Chun et al. | 44/39 | acute AF | NK | Wenxin Keli | Amiodarone | 28 |
| 125 | 2014 | Wang Hui Ying et al. | 30/30 | PAF | dual deficiency of qi and yin | Qiling Formula + Amiodarone | Amiodarone | 60 |
| 126 | 2014 | Kang Xiao Hong et al. | 40/40 | PAF | NK | Shensong Yangxin Capsules + Amiodarone | Amiodarone | 180 |
| 127 | 2014 | Meng Qing Feng et al. | 60/60 | PAF | qi deficiency and blood stasis | Yangxin Dingji Decoction | conventional treatment | NK |
| 128 | 2014 | Jiang Ju Chen et al. | 61/60 | NK | NK | Tongxinluo Capsules + Amiodarone | Amiodarone | 365 |
| 129 | 2014 | Ma Yong Sheng et al. | 40/40 | PAF | NK | Wenxin Keli + conventional treatment | conventional treatment | NK |
| 130 | 2014 | Yu Zhu Xian et al. | 76/76 | PeAF | NK | Shensong Yangxin Capsules + warfarin + irbesartan + Amiodarone | warfarin + irbesartan + Amiodarone | NK |
| 131 | 2014 | Jin De Yun et al. | 22/20 | NK | NK | Wenxin Keli + Amiodarone | Amiodarone | NK |
| 132 | 2014 | Lei Jing Fen et al. | 32/32/32 | NK | NK | Tongxinluo Capsules + aspirin | 1 warfarin, 2 aspirin | NK |
| 133 | 2015 | Liu Su Zhen et al. | 30/30 | PAF | dual deficiency of qi and yin | Wenxin Decoction + propafenone | propafenone | 90 |
| 134 | 2015 | Huang Yong Xiang et al. | 30/30 | NK | NK | Compound Xueshuantong Capsules | conventional treatment | 30 |
| 135 | 2015 | Wang Zong Hua et al. | 64/64 | NK | NK | self-designed cardioversion decoction + verapamil | verapamil | 30 |
| 136 | 2015 | Sun Jian Rong et al. | 32/30 | PeAF | NK | Wenxin Keli + Amiodarone + warfarin + low-molecular-weight heparin | Amiodarone + warfarin + low-molecular-weight heparin | 28 |
| 137 | 2015 | Pan Chao Qun et al. | 45/45 | NK | NK | Yangxin Dingji Formula + metoprolol + warfarin | metoprolol + warfarin | 60 |
| 138 | 2015 | Cao Wen Zhai et al. | 109/111 | NK | NK | Shensong Yangxin Capsules + conventional treatment | conventional treatment | 28 |
| 139 | 2015 | Yang Xing Guo et al. | 120/132 | PeAF | NK | Wenxin Keli + conventional treatment | conventional treatment | NK |
| 140 | 2015 | Hao Yan Hua et al. | 30/30 | PAF | NK | Wenxin Keli + metoprolol | metoprolol | 14 |
| 141 | 2015 | Han Rui et al. | 47/47 | NK | NK | Wenxin Keli + Amiodarone | Amiodarone | NK |
| 142 | 2015 | Chen Da Guo et al. | 32/28 | AF with RVR | NK | Wenxin Keli + Amiodarone | Amiodarone | 28 |
| 143 | 2015 | Lin Bing et al. | 30/29 | PAF | NK | Shensong Yangxin Capsules | propafenone | 90 |
| 144 | 2015 | Cheng Jing et al. | 100/100 | PermAF, PeAF | deficiency of qi and blood + phlegm-stasis obstruction | Yangxin Guicao Decoction + warfarin + metoprolol | warfarin + metoprolol | 180 |
| 145 | 2015 | Li Fen Xia et al. | 56/55 | PAF | NK | Xinsuning Capsules | conventional treatment | NK |
| 146 | 2015 | Wang Xia et al. | 34/34 | NK | NK | Shensong Yangxin Capsules + Amiodarone | Amiodarone | NK |
| 147 | 2015 | Yin Chun Shi et al. | 96/93 | PAF | NK | Wenxin Keli + Amiodarone | Amiodarone | 28 |
| 148 | 2015 | Ma Jun et al. | 100/100 | NK | NK | Shensong Yangxin Capsules + Amiodarone | Amiodarone | 30 |
| 149 | 2016 | Su Jia Bo et al. | 100/100 | PAF | NK | Yangxue Fumai Decoction + propafenone | propafenone | 28 |
| 150 | 2016 | Ma Li et al. | 31/30 | PeAF | NK | Breviscapine Injection + warfarin | warfarin | 14 |
| 151 | 2016 | Wang Guan Hua et al. | 53/53 | PAF, PeAF, PermAF | NK | Shensong Yangxin Capsules + warfarin | warfarin | 84 |
| 152 | 2016 | Wang Xin et al. | 50/50 | acute AF | NK | Wenxin Keli + propafenone | propafenone | 30 |
| 153 | 2016 | Wu Han et al. | 60/60 | NK | NK | Wenxin Keli + Amiodarone | Amiodarone | 60 |
| 154 | 2016 | Li Fa Zhu et al. | 45/45 | PAF | phlegm-stasis obstruction | Shensong Yangxin Capsules + Amiodarone | Amiodarone | 30 |
| 155 | 2016 | Chen Qiang et al. | 31/30 | PAF | NK | Wenxin Keli + metoprolol | metoprolol | 14 |
| 156 | 2016 | Tian Hua Wei et al. | 70/70 | NK | NK | Wenxin Keli + ibutilide fumarate injection | ibutilide fumarate injection | 28 |
| 157 | 2016 | Deng Shui Qing et al. | 46/46 | NK | NK | Wenxin Keli + propafenone | propafenone | 28 |
| 158 | 2016 | Gao De Cai et al. | 33/33 | PAF | NK | Wenxin Keli + metoprolol | metoprolol | 28 |
| 159 | 2016 | Wu Hao et al. | 51/45 | PermAF | NK | Maixuekang Capsules + warfarin | warfarin | NK |
| 160 | 2016 | Jiang Li Li et al. | 63/63 | PAF | NK | Wenxin Keli + Amiodarone + benazepril hydrochloride | Amiodarone + benazepril hydrochloride | 360 |
| 161 | 2016 | Liu Fu Xiang et al. | 80/80 | NK | NK | self-prepared cardioversion decoction + Amiodarone | Amiodarone | 90 |
| 162 | 2016 | Xie Mei et al. | 58/58 | PAF | NK | Wenxin Keli + bisoprolol | bisoprolol | 14 |
| 163 | 2016 | Zhang Rong Zhen et al. | 42/42 | PAF | NK | Wenxin Keli + Amiodarone | Amiodarone | 180 |
| 164 | 2016 | Meng Fan Jie et al. | 30/30 | PAF | NK | Wenxin Keli + conventional treatment | conventional treatment | NK |
| 165 | 2016 | Cao Wen Zhai et al. | 109/111 | PAF, PeAF, PermAF | NK | Wenxin Keli + conventional treatment | conventional treatment | NK |
| 166 | 2016 | Jiang Chao Peng et al. | 50/48 | NK | dual deficiency of qi and yin + blood stasis | Yixinshu Capsules + conventional treatment | conventional treatment | NK |
| 167 | 2016 | Wang Li Nuo et al. | 36/36 | PermAF | NK | Wenxin Keli + conventional treatment | conventional treatment | NK |
| 168 | 2016 | Yang Fu Ping et al. | 50/50 | PAF | NK | Wenxin Keli + atorvastatin | Amiodarone | 60 |
| 169 | 2017 | Yuan Ming et al. | 25/25 | NK | qi deficiency and blood stasis | self-designed Yiqi Huoxue Formula + conventional treatment | conventional treatment | 28 |
| 170 | 2017 | Liu Chun Yan et al. | 38/38 | PAF | qi deficiency and blood stasis | Yiqi Tongmai Decoction + conventional treatment | conventional treatment | 35 |
| 171 | 2017 | Zhang Nan et al. | 31/31 | PAF | NK | Wenxin Keli + Amiodarone | Amiodarone | NK |
| 172 | 2017 | Wang Jing et al. | 50/50 | NK | NK | self-designed cardioversion decoction for atrial fibrillation + deslanoside injection | deslanoside injection | 28 |
| 173 | 2017 | Dang Ya Nan et al. | 75/75 | PAF, PeAF, PermAF | NK | Shensong Yangxin Capsules + warfarin | warfarin | 84 |
| 174 | 2017 | Jin Jie et al. | 33/33 | PeAF | NK | Shensong Yangxin Capsules + Amiodarone | Amiodarone | 28 |
| 175 | 2017 | Huang Xiao Jun et al. | 45/45 | PAF | NK | Shensong Yangxin Capsules + metoprolol | metoprolol | 28 |
| 176 | 2017 | Liu Jun et al. | 34/34 | PAF | qi stagnation and blood stasis + dual deficiency of qi and yin | Dingxin Granules + metoprolol | metoprolol | 28 |
| 177 | 2017 | Jiang Li Fang et al. | 37/35 | NK | NK | Shensong Yangxin Capsules | propafenone | 180 |
| 178 | 2017 | Cai Yan Ping et al. | 55/53 | PAF | NK | Wenxin Keli + Amiodarone + enalapril | Amiodarone + enalapril | 180 |
| 179 | 2017 | Zhuo Zhao Gui et al. | 40/40 | NK | NK | Shensong Yangxin Capsules + Amiodarone | Amiodarone | 180 |
| 180 | 2017 | Zhou Ji Qiang et al. | 102/102 | PAF | NK | Shensong Yangxin Capsules + Amiodarone | conventional treatment | 21 |
| 181 | 2017 | Ni Fei Zhen et al. | 40/40 | NK | phlegm-turbidity obstruction + internal disturbance of phlegm-heat + heart-blood stasis obstruction | Gualou Xiebai Banxia Decoction + Huanglian Wendan Decoction + Xuefu Zhuyu Decoction | metoprolol + aspirin | NK |
| 182 | 2018 | Yu Ping et al. | 40/40 | NK | blood stasis | Xuefu Zhuyu Decoction + dabigatran etexilate capsules | dabigatran etexilate capsules | 28 |
| 183 | 2018 | Chen Cai Xia et al. | 46/46 | PAF | NK | Wenxin Keli + Amiodarone + enalapril | Amiodarone + enalapril | 180 |
| 184 | 2018 | Hu Yu Yue et al. | 48/48 | PAF | NK | Shensong Yangxin Capsules + Amiodarone | Amiodarone + Wenxin Keli | 360 |
| 185 | 2018 | Hu Hao Bing et al. | 36/36 | acute AF | NK | Shensong Yangxin Capsules + Amiodarone | Amiodarone | NK |
| 186 | 2018 | Chen Hui et al. | 42/40 | PAF | dual deficiency of qi and yin | Wenxin Keli + bisoprolol | bisoprolol | 84 |
| 187 | 2018 | He Jian Zhen et al. | 38/38 | PAF | NK | Yangyin Fuli Decoction + Amiodarone + aspirin | Amiodarone + aspirin | 28 |
| 188 | 2018 | Wang Xin Bin et al. | 24/22 | PAF | NK | Wenxin Keli + metoprolol | metoprolol | 180 |
| 189 | 2018 | Yang Mei et al. | 42/42 | PAF | NK | Wenxin Keli + propafenone | propafenone | 56 |
| 190 | 2018 | Wang Jun et al. | 81/81 | PAF | NK | Wenxin Keli + Amiodarone + enalapril | Amiodarone + enalapril | 180 |
| 191 | 2018 | Wang Hong Yan et al. | 75/75 | NK | NK | Wenxin Keli + propafenone | propafenone | 28 |
| 192 | 2018 | Wu Zhi Gang et al. | 52/53 | NK | NK | Wenxin Keli + metoprolol | metoprolol | 56 |
| 193 | 2018 | Wu Gui Ping et al. | 40/40 | PAF | NK | Wenxin Keli + Amiodarone | Amiodarone | 90 |
| 194 | 2018 | Tian Xiao Jie et al. | 20/20 | PermAF | insufficiency of heart yang | Wenxinlü Mixture + conventional treatment | conventional treatment | 28 |
| 195 | 2018 | Zhou Tao et al. | 65/65 | PAF | NK | Compound Danshen Dripping Pills + atorvastatin | atorvastatin | 360 |
| 196 | 2019 | Song Chang Yan et al. | 40/40 | PermAF | phlegm-heat with qi stagnation | self-designed Pinglü Decoction | aspirin + atorvastatin + isosorbide mononitrate | 30 |
| 197 | 2019 | Yu Yan et al. | 19/19 | NK | NK | Wenxin Keli + conventional treatment | conventional treatment | 30 |
| 198 | 2019 | Tang Zhi Hua et al. | 53/53 | PAF | NK | Wenxin Keli + Amiodarone + enalapril | Amiodarone + enalapril | 180 |
| 199 | 2019 | Zhang Yan et al. | 49/49 | NK | NK | Shensong Yangxin Capsules + warfarin | warfarin | 90 |
| 200 | 2019 | Pan Jian Hong et al. | 30/30 | PAF | dual deficiency of qi and yin | Shensong Yangxin Capsules + Amiodarone | Amiodarone | 90 |
| 201 | 2019 | Zhou Ji Zhu et al. | 60/60 | PeAF | NK | Shensong Yangxin Capsules + Amiodarone | Amiodarone | 90 |
| 202 | 2019 | Cheng Yao et al. | 45/45 | NK | NK | Shensong Yangxin Capsules + Amiodarone | Amiodarone + Wenxin Keli | 28 |
| 203 | 2019 | Wang Chao Fan et al. | 30/30 | PermAF | dual deficiency of qi and yin | Dingxin Formula + conventional treatment | conventional treatment | NK |
| 204 | 2019 | Li Jian Hui et al. | 62/62 | NK | NK | Zhigancao Decoction + Amiodarone | Amiodarone | 7 |
| 205 | 2019 | Tian Zhi Qiang et al. | 76/76 | NK | NK | Wenxin Keli + dabigatran etexilate capsules | metoprolol + dabigatran etexilate capsules | 60 |
| 206 | 2019 | Hu Zhu Jun et al. | 40/40 | PAF | NK | Shensong Yangxin Capsules + metoprolol | metoprolol | 180 |
| 207 | 2019 | Chen Hui et al. | 61/55 | PAF | NK | Wenxin Keli + Amiodarone + enalapril | Amiodarone + enalapril | 180 |
| 208 | 2020 | Yan Zhu Qing et al. | 40/40 | PermAF | NK | Jianxin Pinglü Pills + metoprolol + anticoagulants | metoprolol + anticoagulants | 360 |
| 209 | 2020 | Cai Ji Rui et al. | 47/47 | PAF | NK | Ningxinbao Capsules + Amiodarone | Amiodarone | 360 |
| 210 | 2020 | Xie Jian et al. | 33/34 | PAF | NK | Yangxin Dingji Capsules + Amiodarone | Amiodarone | 360 |
| 211 | 2020 | Liu Hong En et al. | 26/26 | PAF | NK | Wenxin Keli + atorvastatin | atorvastatin | 180 |
| 212 | 2020 | Li Ping Ping et al. | 36/36 | PAF | dual deficiency of qi and yin | Wenxin Keli | Amiodarone | 56 |
| 213 | 2020 | Jiang Wei Feng et al. | 54/54 | PAF | NK | Wenxin Keli | dronedarone hydrochloride | 180 |
| 214 | 2020 | Wang Li Xiao et al. | 58/60 | NK | internal disturbance of phlegm-heat | Qingre Fumai Decoction + warfarin + digoxin | warfarin + digoxin | 30 |
| 215 | 2020 | Li Dan Dan et al. | 60/60 | NK | NK | Maixuekang Capsules + aspirin | aspirin | 28 |
| 216 | 2020 | Wang Yu Qi et al. | 32/32 | NK | NK | Tongmai Yangxin Pills + metoprolol + aspirin | metoprolol + aspirin | 28 |
| 217 | 2020 | Liu Yan et al. | 38/38 | PAF | NK | Shensong Yangxin Capsules + bisoprolol | bisoprolol | 180 |
| 218 | 2020 | Tuo Tian et al. | 46/46 | PAF | NK | Shensong Yangxin Capsules + valsartan tablets | valsartan | 84 |
| 219 | 2020 | Gu Zhuan Xia et al. | 75/75 | NK | NK | Shensong Yangxin Capsules + metoprolol | metoprolol | 21 |
| 220 | 2020 | Zhu Lin Lin et al. | 50/50 | NK | NK | Wenxin Keli + metoprolol | metoprolol | 56 |
| 221 | 2021 | Pan Jian Hong et al. | 30/30 | PAF | NK | Shensong Yangxin Capsules + Amiodarone | Amiodarone | 90 |
| 222 | 2021 | Chen Yan et al. | 30/31 | NK | yang deficiency with phlegm coagulation | Yixin Decoction + conventional treatment | conventional treatment | 90 |
| 223 | 2021 | Xie Hai Tao et al. | 45/45 | PeAF | dual deficiency of qi and yin | Zhigancao Decoction + metoprolol + dabigatran etexilate capsules | metoprolol + dabigatran etexilate capsules | 56 |
| 224 | 2021 | Chang Lin et al. | 42/43 | NK | internal disturbance of phlegm-heat | self-designed Qingxin Huatan Decoction + Danhong Injection | Danhong Injection | 14 |
| 225 | 2021 | Xie Xing Hui et al. | 52/52 | NK | NK | self-designed cardioversion decoction + Amiodarone | Amiodarone | 30 |
| 226 | 2021 | Zhao Peng et al. | 20/20 | PAF | NK | Fumai Yangxin Decoction + Amiodarone | Amiodarone | 60 |
| 227 | 2021 | Li Nan et al. | 35/35 | NK | dual deficiency of qi and yin + heart-blood stasis obstruction | Wenxin Keli | metoprolol | 56 |
| 228 | 2021 | Ying Xiao et al. | 36/36 | PAF | qi deficiency with phlegm accumulation | Huatan Dingji Granules + conventional treatment | placebo + conventional treatment | 28 |
| 229 | 2022 | Zhang Fan Zhi et al. | 72/72/71 | PAF | NK | Shensong Yangxin Capsules | 1 blank control, 2 propafenone | 90 |
| 230 | 2023 | Zhang Guo Wei et al. | 30/30 | PeAF | NK | Shensong Yangxin Capsules | anticoagulants + amiodarone and other antiarrhythmic drugs | 180 |
| 231 | 2023 | Ma Xiao Mei et al. | 32/31 | PAF | qi deficiency and blood stasis | self-designed Yiqi Fumai Formula + Amiodarone + dabigatran etexilate capsules | Amiodarone + dabigatran etexilate capsules | 28 |
| 232 | 2024 | Pang Xiao Xuan et al. | 31/31 | PAF | NK | self-designed Yiqi Fumai Formula + conventional treatment | conventional treatment | 90 |
| *Abbreviations: NK, not known. PeAF, Persistent atrial fibrillation. PermAF, Permanent atrial fibrillation. PAF, Paroxysmal atrial fibrillation. CAF, Chronic atrial fibrillation. AF with RVR, Atrial fibrillation with rapid ventricular response. acute AF, Acute atrial fibrillation.* | | | | | | | | |
