# Supplementary material for: Development of a core outcome set for traditional Chinese medicine for atrial fibrillation
Source: Front Pharmacol. 2026 May 7;17:1731405. doi: 10.3389/fphar.2026.1731405 (PMC13216717; doi:10.3389/fphar.2026.1731405)
Supplement: Supplementary file 2 [file DataSheet1.docx]

Supplementary table 1. Search strategy

| 1. PubMed |
| --- |
| ((((traditional Chinese medicine[Title/Abstract]) OR (Chinese patent medicine[Title/Abstract])) OR (TCM[Title/Abstract])) AND (((randomized controlled trial[Title/Abstract]) OR (randomized controlled study[Title/Abstract])) OR (RCT[Title/Abstract]))) AND ((Atrial Fibrillation[Title/Abstract]) OR (Auricular Fibrillation[Title/Abstract])) |
| 1. Web of Science |
| TS=("Atrial Fibrillation" OR "Auricular Fibrillation" OR "Persistent Atrial Fibrillation" OR "Familial Atrial Fibrillation" OR "Paroxysmal Atrial Fibrillation") AND TS=("traditional Chinese medicine" OR "Chinese patent medicine" OR "TCM" OR "CPM") AND TS=("randomized controlled trials" OR "randomized controlled study" OR "randomized controlled trial" OR "randomized study" OR "randomized trial" OR "randomized placebo-controlled study" OR "randomized placebo-controlled trial" OR "RCT”) |
| 1. Cochrane Library |
| #1 (traditional Chinese medicine):ti,ab,kw OR (Chinese patent medicine):ti,ab,kw OR (TCM):ti,ab,kw OR (CPM):ti,ab,kw  #2 (Atrial Fibrillation):ti,ab,kw OR (Auricular Fibrillation):ti,ab,kw OR (Persistent Atrial Fibrillation):ti,ab,kw OR (Familial Atrial Fibrillation):ti,ab,kw OR (Paroxysmal Atrial Fibrillation):ti,ab,kw  #3 (randomized controlled trial):ti,ab,kw OR (randomized controlled study):ti,ab,kw OR (RCT):ti,ab,kw  #4 #1 AND #2 AND #3 |
| 1. Embase |
| ("Atrial Fibrillations" OR "Auricular Fibrillation") AND ("randomized controlled trial" OR "randomized controlled study” OR “RCT”) AND ("traditional Chinese medicine" OR "Chinese patent medicine" OR "TCM" OR "CPM”) |
| 1. CNKI |
| ((TKA%='中医药' OR TKA%='中成药' OR TKA%='中药') AND (TKA%='房颤' OR TKA%='心房颤动’ OR TKA%='心房纤颤’ OR TKA%='心悸’) AND (FT%='随机')) |
| 1. WanFang |
| 主题:(房颤 OR 心房颤动 OR 心房纤颤 OR 心悸) AND 主题:(中医药 OR 中成药 OR 中药) and 全部:(随机) |
| 1. VIP |
| (M=房颤 OR M=心房颤动 OR M=心悸) AND (M=中医药 OR M=中成药 OR M=中药) AND (U=随机) |
| 1. SinoMed |
| ( ( "心房颤动"[常用字段:智能] OR "房颤"[常用字段:智能] OR "心房纤颤"[常用字段:智能] OR "心悸"[常用字段:智能]) AND( "中医药"[常用字段:智能] OR "中成药"[常用字段:智能] OR "中药"[常用字段:智能]) AND( "随机"[常用字段:智能]) ) |
